# Phase II Clinical Trial of Robotic Stereotactic Body Radiosurgery for Metastatic Gynecologic Malignancies

**DOI:** 10.3389/fonc.2012.00181

**Published:** 2012-12-05

**Authors:** Charles A. Kunos, James Brindle, Steven Waggoner, Kristine Zanotti, Kimberly Resnick, Nancy Fusco, Ramon Adams, Robert Debernardo

**Affiliations:** ^1^Department of Radiation Oncology, University Hospitals Case Medical Center and Case Western Reserve University, School of MedicineCleveland, OH, USA; ^2^Division of Gynecologic Oncology, Department of Obstetrics and Gynecology, University Hospitals Case Medical Center and Case Western Reserve University, School of MedicineCleveland, OH, USA

**Keywords:** robotic radiosurgery, Cyberknife, gynecologic cancer

## Abstract

**Background:** Recurrent gynecologic cancers are often difficult to manage without significant morbidity. We conducted a phase II study to assess the safety and the efficacy of ablative robotic stereotactic body radiosurgery (SBRT) in women with metastatic gynecologic cancers. **Methods:** A total of 50 patients with recurrent gynecologic cancer who had single or multiple (≤4) metastases underwent robotic-armed Cyberknife SBRT (24Gy/3 daily doses). Toxicities were graded prospectively by common toxicity criteria for adverse events (version 4.0). SBRT target responses were recorded following RECIST criteria (version 1.0). Rates of clinical benefit for SBRT and non-radiosurgical disease relapse were calculated. Disease-free and overall survivals were estimated by the Kaplan–Meier method and the Cox proportional hazards model was used to control for prognostic variables. **Findings:** SBRT was safely delivered, with 49 (98%) of 50 patients completing three prescribed fractions. The most frequent grade 2 or higher adverse events attributed to SBRT included fatigue (16%), nausea (8%), and diarrhea (4%). One (2%) grade four hyperbilirubinemia occurred. SBRT target response was 96% (48 of 50 patients). A 6-month clinical benefit was recorded in 34 [68% (95% CI, 53.2, 80.1)] patients. No SBRT targeted disease progressed. Non-radiosurgical disease relapse occurred in 31 (62%) patients. Median disease-free survival was 7.8 months (95% CI, 4.0, 11.6). Median overall survival was 20.2 months (95% CI, 10.9, 29.5). **Interpretation:** SBRT safely controlled metastatic gynecologic cancer targets. Given an observed high rate of non-radiosurgical disease relapse, a phase I trial assessing co-administration of SBRT and cytotoxic chemotherapy is underway. **Funding:** Case Comprehensive Cancer Center.

## Introduction

Leading causes of cancer-related death in women worldwide include metastatic and recurrent ovarian, uterine, uterine cervix, and vulvar cancers (Ferlay et al., 2010). When these cancers metastasize after primary surgical, chemotherapy, or radiotherapy, conventional treatment options often are associated with morbidity. These women therefore have an unmet therapeutic need. As many as 40% of women with gynecologic cancer have disease relapse occurring in previously irradiated tissues or occurring near chemotherapy-taxed bone marrow (Kunos et al., 2012a). Because of prior surgical, chemotherapy, and radiation treatment, it is often difficult to use radiotherapy without functional hazard to normal organs. Extracranial stereotactic body radiosurgery (SBRT) permits a non-invasive therapeutic option for women with gynecologic cancer disease relapse.

Stereotactic body radiosurgery offers advantages, when compared to other radiation therapy platforms, both in its accounting of target motion and in its eloquent dosimetry. Indeed, SBRT capitalizes upon on-board image guidance of “pencil beam-sized” portals delivering hypofractionated radiation (Kunos et al., 2008, 2009, 2011, 2012a; Choi et al., 2009; Deodato et al., 2011; Higginson et al., 2011). Except for recognized normal organ tolerances to ablative radiation dose, (Timmerman, 2008) patient eligibility guidelines for SBRT remain controversial in the management of gynecologic cancers. As one example of a SBRT platform, the Cyberknife^®^ system [Accuray (Sunnyvale, CA, USA)] utilizes a linear accelerator mounted to an industrial robotic arm to direct radiation dose, without the use of a rigid frame, precisely at cancer targets while tracking cancer-target motion during the treatment, ultimately achieving submillimeter accuracy (Wilcox and Daskalov, 2007).

This phase II clinical trial was designed to evaluate whether ablative radiation could control metastatic gynecologic cancer with acceptable toxicity levels. Institutional experiences of SBRT alone in patients with gynecologic cancers have shown SBRT to have modest toxicity, (Kunos et al., 2008, 2009, 2012a; Choi et al., 2009; Deodato et al., 2011; Higginson et al., 2011) with radiation dose and number of radiosurgical fractions important determinants of side effects (Timmerman, 2008). As such, we investigated the safety and efficacy of ablative robotic SBRT in this first-reported clinical trial conducted in women with metastatic gynecologic cancers.

## Methods

### Study design and patients

For this clinical trial, patients were included if they were women 18 years of age or older. Patients had an Eastern Cooperative Group performance status of 0, 1, 2, or 3. Patients must have had a single metastasis or metastases (≤4) of a pathologically confirmed gynecologic cancer whose dimensions could be measured by RECIST criteria (version 1.0; Nishino et al., 2010). In this trial, there was no constraint for the total planned radiosurgical target volume. Patients may not have had cryosurgery or radiofrequency ablation in the planned radiosurgical target (as these forms of treatment would obscure SBRT response measurements). Patients must have had adequate pretherapy hematological and renal function. There were no pretherapy limits for the number of prior chemotherapy cycles or radiation dosage. Patients must have stopped anticancer therapies for 4 weeks before SBRT.

Fifty women met these criteria between July 2009, and September 2011. Their demographics, gynecologic cancer type, number of SBRT target metastases, and prior chemotherapies or radiation are cataloged in Table 1. All patients provided written informed consent. University Hospitals of Cleveland and Case Western Reserve University (Cleveland, OH, USA) Institutional Review Board approval was granted for this clinical trial. Oversight for the data and safety monitoring plan was provided by the Case Comprehensive Cancer Center of University Hospitals of Cleveland and Case Western Reserve University.

**Table 1:** **Baseline characteristics of the study patients**.

Characteristic	
Women – no. (%)	50 (100)
**Age – year**	
Median	66
Range	27−82
Race – no. (%)*	
White	41 (82)
Black or African ancestry	6 (12)
Hispanic	3 (6)
**Ecog performance status – no. (%)^†^**	
0	36 (72)
1	7 (14)
2	7 (14)
3	0 (0)
**No. of metastases to be treated by radiosurgery – no. (%)**	
1	14 (28)
2	21 (42)
3	13 (26)
4	2 (4)
≥4	0 (0)
**Histopathology – no. (%)**	
Cervix/vagina squamous cell carcinoma	9 (18)
Endometrial adenocarcinoma	14 (28)
Ovarian adenocarcinoma	25 (50)
Vulvar squamous cell carcinoma	2 (4)
Prior radiation – no. (%)	22 (44)
Inclusive of radiosurgery site	16 (32)
>5000 cGy delivered pretherapy to radiosurgery site	13 (26)
Prior chemotherapy (any) – no. (%)	47 (94)
**No. of prior courses of chemotherapy for metastates – no. (%)**	
0	28 (56)
1	20 (40)
2	2 (4)
Platinum-containing regimen – no. (%)	21 (42)
Taxane-containing regimen – no. (%)	14 (28)
Anthracycline-containing regimen – no. (%)	6 (12)
Topotecan-containing regimen – no. (%)	5 (10)
Gemcitabine-containing regimen – no. (%)	5 (10)

*Race was self-reported.

*^†^The Eastern Cooperative Group (ECOG) performance status reflects individual daily living activities on a scale of 0 (fully active with symptoms) to 5 (dead)*.

*no. = number*.

### Stereotactic body radiosurgery

For radiosurgical planning, patients underwent same-day thorax to mid-thigh non-contrasted contiguous axial computed tomography (CT) high-resolution imaging (voxel 0.98 mm × 0.98 mm × 1 mm, technique: 120 kVp, 450 mAs) and axial ^18^F-deoxyglucose positron emission tomography (^18^F-FDG PET) images acquired in the head-first supine position (voxel: 4 mm × 4 mm× 4 mm) following institutional protocol, (Kunos et al., 2011, 2012a) CT and ^18^F-FDG PET images were imported, digitally overlaid, and co-registered for inverse radiation treatment planning on the MultiPlan 3.5.2 Treatment Planning System (Accuray). Based on these images, we targeted their entire known disease burden as a clinical target volume (CTV) because it included delineated gross tumor volume (GTV) and ^18^F-FDG PET signal outside the GTV. A 3.0 mm margin was added to the CTV for a planning tumor volume (PTV). Normal tissue contours were applied. The radiation prescription was three daily fractions of 8 Gy per fraction totaling 24 Gy (typically prescribed to the 70% isodose line). Fixed tungsten circular collimators (5–60 mm) or a tungsten-copper alloy iris aperture collimated a 6 MV radiation beam. For additional details, the reader is referred to a peer-reviewed, video-complemented algorithm for the three outpatient robotic SBRT treatment sessions (Kunos et al., 2012b).

### Safety assessments

Patients underwent physical examination, hematologic, and renal blood tests as well as baseline CT and ^18^F-FDG PET scans within 28 days before the first SBRT treatment. Physical examinations, CT scans for response, and adverse event assessments [graded 0 (none) to 5 (fatal), according to Common Toxicity Criteria for Adverse Events (version 4.0)] were repeated at 1 and 3 months mandatorily. Thereafter, these evaluations were recommended every 6 months, with additional imaging and blood tests repeated at the option of the treating physician.

### Evaluation of clinical activity and statistical methods

Stereotactic body radiosurgery target responses were recorded following RECIST (Nishino et al., 2010). A rate of clinical benefit for SBRT was calculated [i.e., number of complete (CR) + partial (PR) + stable disease (SD) response for ≥6 months, without new elsewhere progression of disease (PD)]. Local disease relapse was defined as disease progression of the SBRT target(s). Non-radiosurgical target (i.e., disease not targeted by SBRT on this clinical trial, but amenable to subsequent treatment including SBRT) distant disease relapse was scored as disease progression. Time at-risk for disease progression or death was measured from the first date of SBRT. Univariate product-limit estimates [95% confidence interval (C.I.)] for progression-free (i.e., disease relapse and death) and overall survival were calculated utilizing the method of Kaplan and Meier (SPSS 18.0, Chicago, IL, USA; Kaplan and Meier, 1958). Descriptive and graphical statistics were computed using statistical software (SPSS 18.0). For this clinical trial, the number of enrollees (*n* = 50) was selected arbitrarily.

## Results

### Patients

Between July 24, 2009 and September 7, 2011, 50 patients underwent SBRT for metastatic gynecologic cancers. A total of 49 patients (98%) received all three prescribed SBRT treatments. One patient did not complete her third fraction of SBRT due to intractable back pain which prevented her from lying on the treatment table. All 50 patients were included in the SBRT safety analysis. As of the date of data cutoff (April 15, 2012), all patients have completed SBRT and have been followed for 6 months or more unless cancer-related death occurred. The median follow-up for surviving patients was 15 months (range, 1–31 months). Four (8%) patients received adjuvant chemotherapy before their scheduled second confirmatory assessment of their disease status.

**Figure 1:**
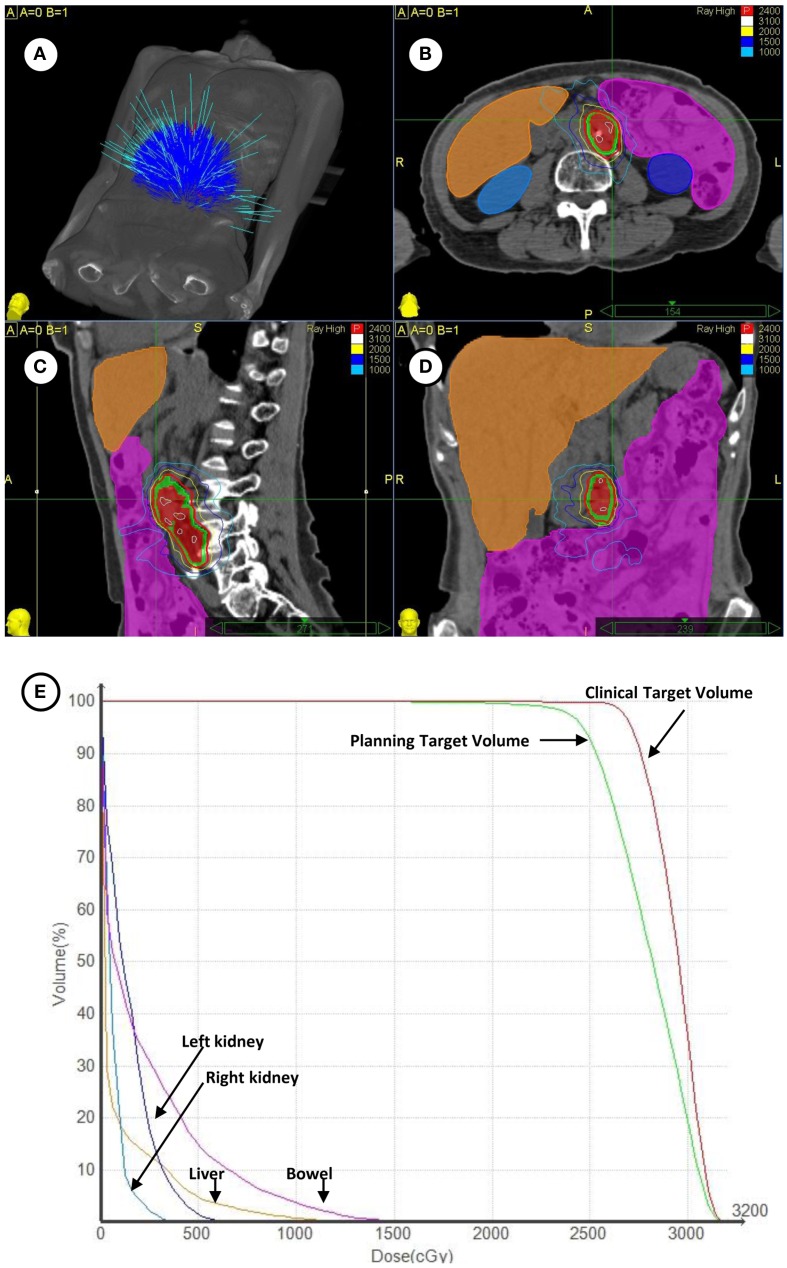
**(A)** Depicted are the 140 treatment beams by the Cyberknife radiosurgery 6 MV accelerator for treatment during the targeting of left-sided para-aortic lymph nodes. **(B–D)** Depicted are axial, coronal, and sagittal projections of radiosurgical treatment. The ^18^F-FDG PET/CT-derived clinical target volume (red shaded volume) and 3 mm expanded planning tumor volume (green shaded volume) are contoured. The 24 Gy prescription isodose is highlighted in red and a 10 Gy isodose is outlined in light blue. Contouring of the bowel (magenta), liver (orange), right kidney (light blue), and left kidney (dark blue) is shown. **(E)** Plotted are corresponding radiation dose-volume histograms for clinical targets and organs at-risk.

Patients with metastatic ovarian (50%), uterine (28%), uterine cervix (18%), and vulvar (4%) cancers were enrolled on this clinical trial (Table 1). Overall, 22 patients (44%) had received prior radiation and 47 patients (94%) had received prior chemotherapy for both initial therapies and recurrent disease before SBRT. A total of 29 patients (58%) received SBRT treatment as first-line therapy for metastatic gynecologic cancer disease. No patient had received SBRT prior to clinical trial enrollment. SBRT sites of treatment and its treatment parameters are listed in Table 2. SBRT was used for lymph node sites of metastatic disease (including para-aortic, pelvic, and groin nodes) in 34 of 50 patients (68%). The median SBRT PTV that encompassed the entirety of known CT and ^18^F-FDG PET identified targets was 68 cm^3^ (range: 4–613 cm^3^; Table 2). An example of SBRT treatment is illustrated in Figure 1.

**Table 2:** **Stereotactic body radiosurgery sites of treatment and treatment parameters**.

**TREATMENT SITE – NO. (%)**	
Para-aortic lymph nodes	19 (38%)
Pelvis lymph nodes/pelvis soft tissue site	14 (28%)
Upper abdomen (excluding para-aortic lymph nodes, liver)	2 (4%)
Liver	8 (16%)
Lung	4 (8%)
Thoracolumbar spine	2 (4%)
Groin lymph nodes/perineal soft tissue site	1 (2%)
**TREATMENT PARAMETERS**	
Median prescription dose (dose × fractions)	2400 cGy (800 cGy × 3)
Median prescription isodose (25–75% quartile)	70% (70−80%)
Radiosurgical tracking
By gold seed fiducials – no. (%)	44 (88%)
By bony spine landmarks – no. (%)	6 (12%)
Radiosurgical beam collimation	
By fixed collimator – no. (%)	23 (46%)
By iris collimator – no. (%)	27 (54%)
Synchrony motion tracking – no. (%)	25 (50%)
Median planning tumor volume (25–75% quartile)	68 cm^3^ (29 −124 cm^3^)

*no. = number*.

### Safety

Table 3 lists all types (i.e., any early ≤30 day or late >30 day) and frequencies of at least possibly SBRT-related adverse events among all 50 patients. The most frequent adverse events were grade 1 or 2 fatigue (20%) and grade 1 or 2 nausea (12%). Fatigue and nausea resolved spontaneously in all nine patients by the 30-day posttherapy physician assessment. The incidence of grade 3 or grade 4 possible SBRT-related non-hematological toxicities was 6%; these events included non-infectious diarrhea, enterovaginal fistula, and hyperbilirubinemia. Grade 3 or grade 4 neutropenias, thrombocytopenias, or anemias attributable to SBRT were not observed within 30 days posttherapy. One death (2%) occurred within 30 days of SBRT due to non-radiosurgical target disease progression resulting in end-organ failure. Among 16 women who had their SBRT treatment overlap their prior external beam radiation therapy site, a single (6%) grade 3 diarrhea adverse event was observed.

**Table 3:** **Stereotactic body radiosurgery-related worst grade toxicities**.

Adverse event	Grade			
	1	2	3	4
**Cardiovascular**
Lymphedema	0	1	0	0
**Constitutional symptoms**
Fatigue	2	8	0	0
Anemia	0	1	0	0
**Gastrointestinal**
Anorexia	1	1	0	0
Constipation	1	0	0	0
Diarrhea	1	1	1	0
Abdominal bloating	1	0	0	0
Gastritis	1	0	0	0
Nausea	2	4	0	0
Vomiting	0	1	0	0
**Genitourinary**
Urethra injury	0	1	0	0
Ureteral obstruction	0	1	0	0
Fistula – vagina	0	0	1	0
**Infection**
Febrile Neutropenia	0	0	0	0
Neutropenia	0	0	0	0
**Metabolic/laboratory**
Liver dysfunction – clinical	0	1	0	0
International normalized ratio (INR) of prothrombin time	1	0	0	0
Hyperbilirubinemia	0	0	0	1
Thrombocytopenia	0	0	0	0
**Pain**
Abdominopelvic pain	0	5	0	0
Chest Wall arthralgia/myalgia	3	0	0	0
Total	13	25	2	1

### Efficacy

The SBRT target response rate (target CR + target PR) was 96% (48 of 50 patients). A confirmed SBRT target CR, meaning disappearance of all target and non-target lesions and no new evidence of disease progression, occurred in a single patient [1 (2%) of 50 patients]. Two of the 50 patients (4%) had SBRT target stable disease; one of these patients with a treated lumbar vertebral body metastasis died within 4 weeks of SBRT due to non-radiosurgical lung and marrow disease progression. No local disease relapses were recorded in SBRT targets. However, non-radiosurgical target distant disease relapse occurred in 31 patients (62%). In these 31 patients, the most frequent new distant sites of disease were the lungs [12 (39%)], abdomen, or pelvis [12 (39%)], liver [4 (13%)], bone [2 (6%)], and inguinal lymph nodes [1 (3%)]. The medial interval to distant disease relapse calculated from the first date of SBRT was 5 months (range, 1–16 months). As such, and excluding the one patient with lumbar vertebral disease, the best overall response rate by RECIST was 61% (30 of 49 patients). The rate of clinical benefit (i.e., 6-month CR + PR + SD without PD) was 68% (34 of 50 patients, 95% CI, 53%, 80%).

As of this writing, 24 patients (48%) have died. Eighty-three percent of deaths (20 of 24) were attributable to non-radiosurgical metastatic gynecologic cancer disease progression. The median progression-free survival was 7.8 months (95% CI, 4.0, 11.6). The median overall survival was 20.2 months (95% CI, 10.9, 29.5).

A total of 4 of 50 patients (8%) received adjuvant chemotherapy before second confirmatory assessment of their disease status. Patients given chemotherapy received a median of four cycles of carboplatin-containing chemotherapy regimen. The combination of SBRT and chemotherapy led to a progression-free survival of 8.1 months (95% CI, 3.4, 12.8). A statistical comparison of an SBRT treatment alone and SBRT plus chemotherapy treatment was not done due to small sample size in the latter cohort.

## Discussion

This phase II clinical trial showed that SBRT effectively controls metastatic disease at a high rate with minimal toxicity in patients with metastatic gynecologic cancers.

Contrary to the typical phase II efficacy end point of overall rate of response, we chose the rate of clinical benefit as the primary end point. Clinical benefit (i.e., number of CR + PR + SD responses for ≥6 months, without new elsewhere PD) was chosen on the presumption that radiosurgery can only exert measurable cytotoxic effects on targeted disease. Radiosurgery cannot be held accountable to control disease undetected by CT and ^18^F-FDG PET imaging. For this reason, objective responses (complete or partial) or disease stabilization were regarded clinically meaningful in assessing the antitumor activity of SBRT. Progression of disease elsewhere in the body shortly after SBRT might signal (1) progression of already-present occult disease, or (2) inability of SBRT to control targeted disease prior to disease dissemination.

Stereotactic body radiosurgery has demonstrated activity against gynecologic cancer at various ablative doses and schedules (Kunos et al., 2008, 2009, 2012a; Choi et al., 2009; Deodato et al., 2011; Higginson et al., 2011). In our study, 24 Gy divided into three consecutive 8 Gy daily fractions were given to take radiobiological advantage of relative radiosensitivity of gynecologic malignancies. The SBRT target rate of response (96%) is in accord with rates described in previous studies of SBRT for metastatic gynecologic cancer (range, 67–79%; Kunos et al., 2008, 2009, 2012a; Choi et al., 2009; Deodato et al., 2011; Higginson et al., 2011).

Our observation that 62% of patients eventually develop distant disease progression after undergoing SBRT attests to the need of concurrent chemotherapy co-administration. Too few patients received chemotherapy in close proximity to dose of SBRT to comment on the safe co-administration of cytotoxic chemotherapies. However, we do suggest cautiously that SBRT may contribute to a lengthened progression-free survival. In some previously reported studies, patients with metastatic ovarian, uterine, uterine cervix, and vulvar cancers have a median progression-free survival of 3 months (range, 2–4 months) and overall survival of 9 months (range, 6–15 months; Long et al., 2005; Dizon et al., 2009; Witteveen et al., 2009; De Geest et al., 2010). In our very heterogeneous patient population undergoing a variety of pretreatment radiation and chemotherapy regimens, we observed a progression-free survival of 7.8 months and overall survival of 20.2 months. We do not presume that SBRT was the only contributor to these cancer-related outcomes, but such data might signal a possible therapeutic gain of sterilizing as much known disease as possible at the time of SBRT. Information on the durability of SBRT response would strengthen this claim. As of this writing, such data is not sufficiently mature. A phase I clinical trial testing SBRT and carboplatin – gemcitabine combination that incorporates such information is underway by our research team.

Stereotactic body radiosurgery was well tolerated in this study despite a heavily pretreated group of patients. Any grade hematological (8%) and grade 3 or 4 gastrointestinal (12%) toxicities observed in our study compare favorably to hematological (19% neutropenia, 9% anemia) and gastrointestinal (11%) of intensity-modulated radiation therapy in the abdomen and pelvis (Brixey et al., 2002; Mundt et al., 2003).

Strengths of our study include a contemporary study population drawn among women with common metastatic gynecologic cancers. SBRT was performed by a specialized radiation therapy team led by experienced radiation oncologist and gynecologic oncologists. Weaknesses include insufficient stratification for gynecologic cancer type, which hampers our appraisal of clinical benefit and progression-free survival. Moreover, the study did not control for prior therapies and considerable variability is identified in the study population. The study also could be strengthened by longer-term follow-up for the sequelae of treatment, durability of SBRT target response, and cancer-related outcome.

Our results indicate that SBRT safely delivers effective ablative radiation dose to metastatic sites of gynecologic cancer. The therapy can be administered with minimal toxicity even in a group of heavily pretreated patients. While highly effective at treating targeted lesions, many of these patients develop progressive disease. An improved understanding of the safety and efficacy of SBRT in conjunction with cytotoxic chemotherapy is of interest and is currently under investigation.

### Panel: Research in context

#### Systematic review

Our manuscript reports the first phase II clinical trial of robotic SBRT conducted in women with metastatic gynecological cancers. We searched PubMed with the terms “radiosurgery,” “gynecologic cancer,” and “clinical trial” for publications between January 1, 1999, and June 1, 2012. We broadened our publication search to include radiosurgery conducted for the treatment of gynecologic tumors. Single institution cohort studies of radiosurgery for gynecological cancers were culled to put this research in context (Kunos et al., 2008, 2009, 2012a; Choi et al., 2009; Deodato et al., 2011; Higginson et al., 2011). The radiation dose delivery and schedule vary considerably among these single institution cohort studies, obscuring the totality of evidence for gynecologic cancer disease control.

#### Interpretation

Our first-ever phase II clinical trial establishes clinical benefit of a standardized (8 Gy × 3 consecutive day) robotic SBRT treatment among 50 women with metastatic gynecological cancers. Based on these findings, clinicians may consider SBRT a new treatment option in this disease setting. Clinical trials incorporating SBRT and co-administered chemotherapy are underway.

## Author Contributions

Charles A. Kunos, James Brindle, Steven Waggoner, Kristine Zanotti, Kimberly Resnick, Nancy Fusco, Ramon Adams, and Robert Debernardo all made substantial contributions to the conception of this clinical trial, its design, data collection and the drafting and writing of this manuscript. This manuscript has been seen, read, and agreed upon in its content by all designated authors.

## Conflict of Interest Statement

The authors declare that the research was conducted in the absence of any commercial or financial relationships that could be construed as a potential conflict of interest.
